# Sustained remission of symptoms and improved health-related quality of life in patients with cryopyrin-associated periodic syndrome treated with canakinumab: results of a double-blind placebo-controlled randomized withdrawal study

**DOI:** 10.1186/ar3535

**Published:** 2011-12-09

**Authors:** Isabelle Koné-Paut, Helen J Lachmann, Jasmin B Kuemmerle-Deschner, Eric Hachulla, Kieron S Leslie, Richard Mouy, Alberto Ferreira, Karine Lheritier, Neha Patel, Ralph Preiss, Philip N Hawkins

**Affiliations:** 1 Centre de Référence des Maladies Autoinflammatoires, Hôpital Kremlin Bicetre, Paris University of Medicine, Le Kremlin Bicetre, Paris, France; 2 UCL Medical School, Gower Street, London, WC1E 6BT, UK; 3 Division of Pediatric Rheumatology, Department of Pediatrics, University Hospital Tübingen, Hoppe-Seyler-Straße 1, 72076 Tuebingen, Germany; 4 Department of Internal Medicine, Claude Huriez Hospital, University of Lille, Lille Cedex, France; 5 University of California at San Francisco, San Francisco, CA 94143, USA; 6 Unité de Rhumatologie Pédiatrique, Hôpital Necker-Enfants Malades, 149 rue de Sèvres, 75015 Paris, France; 7 Novartis Pharma AG, CH-4002, Basel, Switzerland; 8 Novartis Pharmaceuticals Corporation, One Health Plaza, East Hanover, NJ 07936-1080, USA

## Abstract

**Abstract:**

**Introduction**

To assess the effect of canakinumab, a fully human anti-interleukin-1β antibody, on symptoms and health-related quality of life (HRQoL) in patients with cryopyrin-associated periodic syndrome (CAPS).

**Methods**

In this 48-week, phase 3 study, patients with CAPS received canakinumab 150 mg subcutaneously at 8-week intervals. All patients (*n *= 35) received canakinumab during weeks 1 through 8; weeks 9 through 24 constituted a double-blind placebo-controlled withdrawal phase, and weeks 24 through 48 constituted an open-label phase in which all patients received canakinumab. Patient and physician assessments of symptoms, levels of inflammatory markers, and HRQoL were performed.

**Results**

Rapid symptom remission was achieved, with 89% of patients having no or minimal disease activity on day 8. Responses were sustained in patients receiving 8-weekly canakinumab. Responses were lost during the placebo-controlled phase in the placebo group and were regained on resuming canakinumab therapy in the open-label phase. Clinical responses were accompanied by decreases in serum levels of C-reactive protein, serum amyloid A protein, and interleukin-6. HRQoL scores at baseline were considerably below those of the general population. Improvements in all 36-item Short-Form Health Survey (SF-36) domain scores were evident by day 8. Scores approached or exceeded those of the general U.S. population by week 8 and remained stable during canakinumab therapy. Improvements in bodily pain and role-physical were particularly marked, increasing by more than 25 points from baseline to week 8. Therapy was generally well tolerated.

**Conclusions**

Canakinumab, 150 mg, 8-weekly, induced rapid and sustained remission of symptoms in patients with CAPS, accompanied by substantial improvements in HRQoL.

**Trial registration:**

Clintrials.gov NCT00465985

## Introduction

Cryopyrin-associated periodic syndrome (CAPS) is one of the genetic autoinflammatory disorders that are characterized by recurrent bouts of systemic inflammation, resulting in fever, rash, and joint pain [1,2]. Most of these disorders are very rare; CAPS has an estimated prevalence of approximately 1 per million, and even the most common one, familial Mediterranean fever, affects only approximately 100,000 people worldwide. Identification of the genes involved in each disorder has helped to explain why the various conditions have similar manifestations. They all appear to result, directly or indirectly, in overproduction of interleukin-1β (IL-1β), a key pro-inflammatory cytokine that regulates innate immune responses [1,2].

CAPS comprises a spectrum of disease from the mildest form, familial cold autoinflammatory syndrome (FCAS), through Muckle-Wells syndrome (MWS), to the most severe form, chronic infantile neurologic cutaneous and articular syndrome (CINCA), also known as neonatal-onset multisystem inflammatory disease (NOMID). CAPS-related symptoms can have a major impact on a patient's quality of life [3], which can be further affected by delayed diagnosis and inappropriate treatment because of poor recognition of this rare disease by healthcare professionals. Identification of the mutations involved in each of the disorders has helped establish FCAS, MWS, and NOMID as different forms of a single disease. All three disorders are associated with mutations in the *NLRP3 *gene. This encodes NALP3, a key component of the inflammasome complex that regulates the production of IL-1β [1,2]. The mutations present in patients with CAPS lead directly to overproduction of IL-1β; in one study IL-β levels were found to be approximately fivefold higher in patients with CAPS than in healthy individuals [4]. An open-label phase 2 study has shown that canakinumab--which binds selectively to IL-1β, thus potently inhibiting its activity--produces rapid, complete, and sustained responses in adults and children with CAPS [5]. Furthermore, a double-blind, placebo-controlled, randomized withdrawal study has shown that 8-weekly administration of canakinumab to patients with CAPS produces sustained remission of symptoms [6].

Here we report further data from the double-blind, placebo-controlled, randomized withdrawal study, concerning the impact of canakinumab therapy on the individual symptoms of CAPS and on health-related quality of life (HRQoL).

## Materials and methods

### Study design

The study was approved by local independent ethics committees and was conducted in accordance with the ethical principles laid down in the Declaration of Helskini. As reported elsewhere [6], the study consisted of three parts (Additional file 1: Supplementary Figure 1). In part 1, all patients received open-label treatment with a single dose of canakinumab to assess response during the following 8 weeks. Part 2 was a double-blind withdrawal period, in which patients who showed a complete response in part 1 were randomly assigned to receive canakinumab or placebo every 8 weeks for up to 24 weeks. At the end of part 2 or on relapse, patients immediately entered part 3, an open-label treatment period in which they received canakinumab every 8 weeks for a minimum of 16 weeks to make a total study duration of 48 weeks. Canakinumab was administered at a dose of 150 mg [or 2 mg/kg body weight for patients ≤40 kg]. This clinical trial was registered with http://www.clinicaltrials.gov (registration number: NCT00465985).

### Patients

The study enrolled patients aged 4 to 75 years with CAPS associated with an *NLRP3 *mutation. Patients had to have a body weight of ≥ 15 kg but < 100 kg, and all patients entering the study or their parents gave written informed consent. Details of exclusion criteria are described elsewhere [6].

### Assessments of disease activity, definitions, and outcome measures

At screening and follow-up visits, physicians assessed global disease activity and each of the following symptoms by using a 5-point scale (absent, minimal, mild, moderate, or severe): urticarial skin rash, arthralgia, myalgia, headache/migraine, conjunctivitis, fatigue or malaise, and other symptoms related or unrelated to CAPS. Blood samples were collected at screening and specified time points for measurement of concentrations of C-reactive protein (CRP), serum amyloid-A protein (SAA), and interleukin-6 (IL-6), and for assessment of hematologic and biochemical markers.

Patients performed a global assessment of their symptoms (rating whether their symptoms were absent, minimal, mild, moderate, or severe) and assessments of the following individual symptoms [assessing whether they had any of the following symptoms and rating symptoms by using a 5-point scale (absent, minimal, mild, moderate, or severe): fever/chills, skin rash, joint/muscle pain, eye discomfort/redness, fatigue, headache, and other symptoms]. Scores were recorded daily for days 1 through 15 of part 1 and weekly thereafter. Adverse events (AEs) were recorded throughout the study, and patients were asked at each study visit whether they had experienced injection-site reactions.

Complete response to canakinumab was defined as a physician global assessment of disease activity that was minimal or absent, with skin rash that was minimal or absent, and serum values of CRP and/or SAA in the normal range. Patients were eligible for entry into part 2 if they had a complete response to canakinumab by day 15 without relapse by week 8. The upper limit of normal (ULN) for CRP and SAA was 10 mg/L.

### Assessment of health-related quality of life

HRQoL was assessed at baseline and at specified time points, by using the following patient-reported outcome instruments in adults (that is, older than 17 years): the functional assessment of chronic illness therapy-fatigue (FACIT-F) [7,8]; the 36-item Short Form Health Survey (SF-36) standard version [9,10]; and the health assessment questionnaire (HAQ) [11]; and the Child Health Questionnaire, Parent Form 28 (CHQ-PF28) in pediatric patients (that is, younger than 17 years) [12]. The FACIT-F questionnaire evaluates general health status, with fatigue as a key component. It is scored from 0 to 52, with a higher score indicating less fatigue. SF-36 is a 36-item questionnaire that measures the impact of disease on overall quality of life and consists of eight individual domains that can be grouped to derive a physical-component summary score (domain names: physical functioning, PF; role-physical, RF; bodily pain, BP; and general health, GH) and a mental-component summary score (domain names: vitality, VT; social functioning, SF; role-emotional, RE; and mental health, MH). Scores range from 0 to 100, with 0 representing the worst possible health, and 100 representing perfect health. The HAQ assesses a patient's physical ability, functional status, and quality of life through 20 questions concerning difficulty in performing eight common activities of daily living. Patients choose from four response categories with scores of 0 to 3, ranging from "without any difficulty" (0) to "unable to do" (3). The CHQ-PF28 is designed for use in children aged 5 to 17 years and is completed by the parent or guardian. This instrument rates HRQoL on a scale from 0 to 100, with values greater than 50 indicating a better HRQoL than that of the normal child in the United States.

### Data analysis

Analysis of data for clinical response was based on an intent-to-treat population including all patients who received at least one dose of study drug, entering each part of the study. Data for CRP and SAA were analyzed for all patients entering part 2 of the study. Data for IL-6 and HRQoL were analyzed for all patients with evaluable data. Change from baseline for FACIT score, SF-36 PCS, SF-36 MCS, and HAQ score were analyzed by using *t *tests. As these were secondary end points, no adjustments for multiplicity were done in the analyses.

## Results

### Patients and disease characteristics

As reported previously [6], 35 patients enrolled in part 1 of the study; 34 (97.1%) achieved a complete response to canakinumab at the end of part 1 (see Additional file 1: Supplementary Figure 2). Of these, 31 patients entered part 2. Responses were maintained during part 2 in all 15 patients (100%) randomized to canakinumab, after which they entered part 3. Of the 16 patients randomized to placebo, 13 (81.3%) relapsed during part 2 and so proceeded early to part 3. All 31 patients who entered part 2 also entered part 3. Of these, 29 (93.5%) completed the study: one patient withdrew considering the therapeutic response to be unsatisfactory, and a second patient withdrew owing to an AE.

As described previously [6], the study included five pediatric patients (17 years or younger), and approximately two thirds were girls (also see Additional file 1: Supplementary table). The study was designed to enroll patients with MWS (representing mid-spectrum CAPS), but some patients had more-severe disease (overlapping MWS/NOMID). Approximately half of patients (*n *= 17; 48.6%) had previously received anakinra, and nine (25.7%) had previously received canakinumab; 11 patients had not previously received anti-IL-1β therapy.

Medical conditions probably related to CAPS were noted at baseline in many patients, including deafness/impaired hearing (*n *= 26; 74.3%), arthralgia (*n *= 31; 88.6%), conjunctivitis (*n *= 31; 88.6%), urticarial skin rash (*n *= 29; 82.9%), headache (*n *= 25; 71.4%), fatigue (*n *= 24; 68.6%), myalgia (*n *= 19; 54.3%), pyrexia (*n *= 17; 48.6%), depression (*n *= 7; 20.0%), uveitis (*n *= 4; 11.4%), and amyloidosis (*n *= 2; 5.7%). According to physicians, most patients had moderate or severe disease activity (Table 1).

**Table 1:** Severity of CAPS-related symptoms at baseline

Symptom severity *n *(%)	Physician global assessment *n *= 35	Patient global assessment *n *= 31	Physician assessment					
			
			Skin rash *n *= 35	Arthralgia *n *= 35	Myalgia *n *= 35	Headache/migraine *n *= 35	Conjunctivitis *n *= 35	Fatigue/migraine *n *= 35
Absent	0	4 (12.9)	4 (11.4)	10 (28.6)	15 (42.9)	15 (42.9)	5 (14.3)	4 (11.4)
Minimal	2 (5.7)	6 (19.4)	6 (17.1)	5 (14.3)	4 (11.4)	2 (5.7)	8 (22.9)	2 (5.7)
Mild	7 (20.0)	8 (25.8)	9 (25.7)	10 (28.6)	8 (22.9)	9 (25.7)	11 (31.4)	7 (20.0)
Moderate	22 (62.9)	9 (29.0)	15 (42.9)	7 (20.0)	8 (22.9)	3 (8.6)	8 (22.9)	14 (40.0)
Severe	4 (11.4)	4 (12.9)	1 (2.9)	3 (8.6)	0	6 (17.1)	3 (8.6)	8 (22.9)

### Response to therapy

#### Clinical response

**Figure 1:**
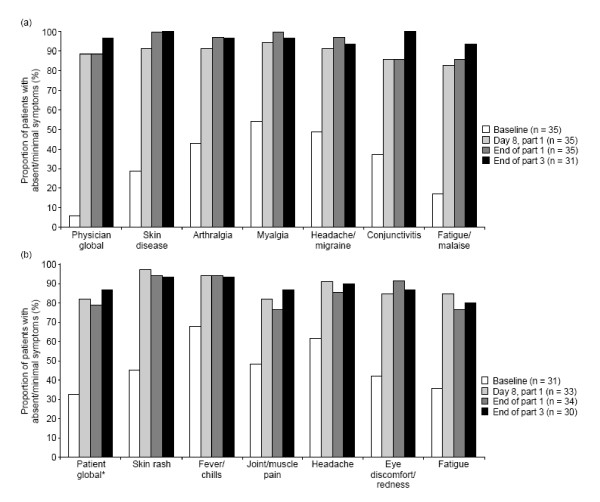
**Physicians' (a) and patients' (b) assessments^a ^of disease activity and individual symptoms over the study**. ^a^Global disease activity and symptoms were assessed by using a 5-point scale. *n = 33 for end of part 1.

A rapid response to canakinumab was seen in part 1, according to physician and patient assessments. By day 8 (the first assessment), physicians assessed disease activity as absent or minimal in 31 (88.6%) patients, and this response was maintained at the end of part 1 (Figure 1a). Similarly, by day 8, 27 (81.8%) patients rated their symptoms as being absent or minimal, and this response was maintained at the end of part 1(Figure 1b).

Ratings for individual symptoms related to CAPS also showed a rapid improvement in response to canakinumab therapy during part 1. By day 8, physicians rated symptoms of arthralgia, myalgia, headache/migraine, and skin rash to be minimal or absent in more than 90% of patients, and conjunctivitis and fatigue were absent or minimal in more than 80% of patients (Figure 1a). Responses were maintained during part 1, with symptoms being rated as absent or minimal at the end of part 1, in more than 95% of patients, for arthralgia, myalgia, headache/migraine, and skin rash, and in more than 85% of patients for conjunctivitis and fatigue/malaise. Similarly, more than 80% of patients rated their individual symptoms as being absent or minimal by day 8, and response rates were similar at the end of part 1 ( 1b).

During part 2 of the study, responses were maintained in patients randomized to canakinumab, according to physician and patient global assessments; disease activity was rated as absent or minimal in 100% of patients, according to the physician assessment and in 66.7% of patients according to patient assessments. In addition, physicians rated individual CAPS-related symptoms as absent or minimal in more than 85% of patients at the end of part 2. By contrast, treatment responses were lost during the course of part 2 in patients randomized to placebo; at the end of part 2, physicians rated disease activity as absent or minimal in four (25%) patients in the placebo group, and symptoms as absent or minimal in four (25%) patients for fatigue/malaise, eight (50.0%) for conjunctivitis and skin rash, 10 (62.5%) for headache/migraine, and 11 (68.8%) for arthralgia and myalgia. On resuming canakinumab therapy in part 3, patients randomized to placebo in part 2 again showed remission of symptoms: at week 8 in part 3, individual symptoms were rated as absent or minimal in more than 85% of patients, and physicians rated all patients as having no or minimal disease activity. At the end of part 3, physicians rated disease activity as absent or minimal in 30 (96.8%) patients, and 26 patients (86.7%) rated their symptoms as absent or minimal.

#### Suppression of inflammation

**Figure 2:**
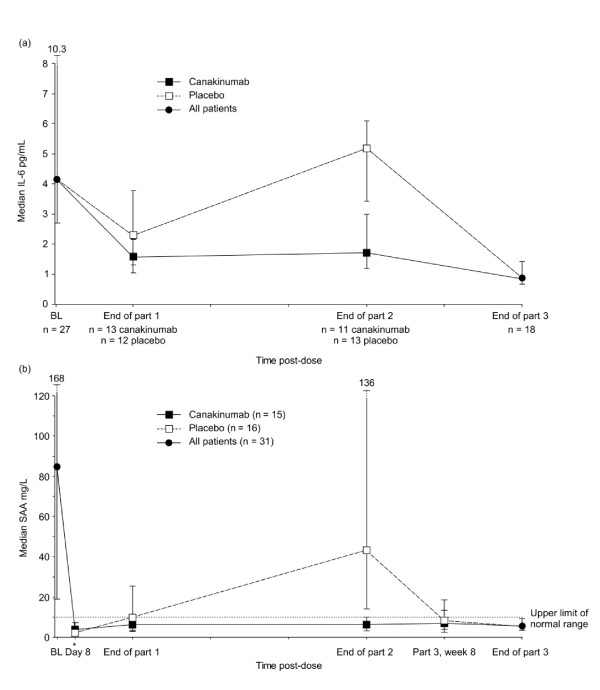
**Interleukin (IL)-6 (a) and serum amyloid A (SAA) levels (b) over the course of the study by treatment group**.^a ^Data are presented as median (interquartile range) *1 week after the start of part 1. Upper limit of normal range for SAA was 10 mg/L.

Median IL-6 levels decreased over the course of part 1 from 4.1 pg/ml at baseline to 1.6 pg/ml (canakinumab group) and 2.3 pg/ml (placebo group) at the end of part 1 (Figure 2a). In the canakinumab group, median IL-6 levels remained low at the end of part 2 (1.7 pg/ml) and at the end of part 3. In contrast, in the placebo group, median IL-6 levels increased over part 2 (after receiving placebo) to 5.2 pg/ml at the end of part 2 and decreased after resumption of canakinumab therapy in part 3. As reported previously, median CRP levels were significantly elevated above ULN at baseline, normalized at the end of part 1, and remained below the ULN for the canakinumab group throughout the study [6]. Median SAA levels changed similarly over the course of the study (Figure 2b). In the placebo group, median CRP and SAA levels increased over the course of part 2, but decreased to below the ULN in part 3 after resuming canakinumab. Abnormalities in the hematologic parameters were not associated with any increased incidence or specific type of adverse events.

#### Impact on HRQoL

At baseline, scores for HRQoL for adults were indicative of a reduced quality of life compared with the general U.S. population (Table 2). Over the course of part 1, the mean FACIT-F score increased significantly from 27.4 at baseline to 40.6 at the end of part 1, an increase of 13.5 (*P *< 0.05), and was 39.5 at the end of part 3. Thus after treatment with canakinumab, the FACIT-F score approached that of the general U.S. population (43.6). Similarly, the mean SF-36 physical-component summary (PCS) score increased from 41.0 at baseline to 51.0 at the end of part 1, an increase of 9.5 (*P *< 0.05), and was 48.5 at the end of part 3. These values correspond approximately to those of the general U.S. population (that is, 50). The mean SF-36 mental-component summary (MCS) score at baseline was slightly higher than for the SF-36 PCS score and showed a smaller but still significant increase over the course of the study from 43.1 at baseline to 47.3 at the end of part 1, up to 48.9 at the end of part 3, an increase of 6.3 (*P *< 0.05) from baseline. As for the FACIT-F and SF-36 PCS, the SF-36 MCS score at the end of the study approached that of the general population (that is, 50). Over the course of the study, the mean HAQ score decreased from 0.41 at baseline to 0.17 at the end of part 1 and 0.27 at the end of the study, indicating a reduction in functional disability.

**Table 2:** HRQoL scores for adults over the course of the study

	**U.S. general population **[8,9]	*N*	Baseline	*n*	End of part 1	Change from baseline at end of part 1	*n*	End of part 3	Change from baseline at end of part 3
FACIT-F	43.6	26	27.4 ± 13.0	24	40.6 ± 12.0	13.5^a^	26	39.5 ± 14.7	12.2^a^
SF-36 PCS	50.0	23	41.0 ± 9.6	20	51.0 ± 8.3	9.5^a^	22	48.5 ± 12.6	8.2^a^
SF-36 MCS	50.0	23	43.1 ± 12.3	20	47.3 ± 13.4	3.6	22	48.9 ± 12.4	6.3^a^
HAQ	NA	26	0.41 ± 0.63	25	0.17 ± 0.40	-0.26^a^	26	0.27 ± 0.45	-0.14

Values are expressed as mean ± SD. ^a^p value < 0.05. FACIT-F, functional assessment of chronic illness therapy- fatigue; HAQ, health assessment questionnaire; MCS, mental component summary; NA, not available; PCS, physical component summary; SF-36, 36-item Short-Form Health Survey.

**Figure 3:**
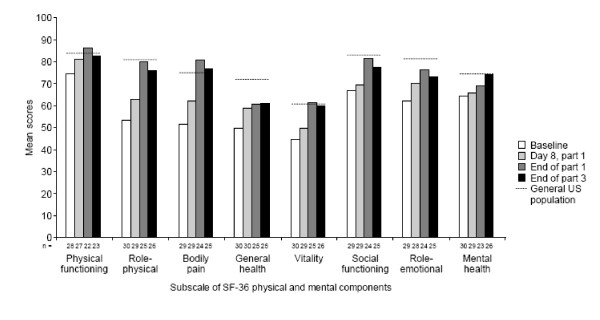
**SF-36^a ^domain scores at baseline and at the ends of part 1 and 3 in adults**. ^a^Data are presented as the mean.

At baseline, mean scores for all SF-36 individual domains were lower than for the U.S. general population, and the difference was particularly marked (> 20 points difference) for role-physical, bodily pain, and general health (Figure 3). Improvements in mean scores were evident for all domains by day 8, the first assessment, and were particularly marked (> 9 points) for role-physical, bodily pain, and general health. Further improvements were evident over the rest of part 1 of the study, with mean scores approaching or exceeding those of the general U.S. population for physical functioning, role-physical, bodily pain, vitality, and social functioning at the end of part 1. The change from baseline in mean score at the end of part 1 (for patients who remained in study at the end of part 1) exceeded 10 points for all domains except mental health and exceeded 25 points for role-physical (26.7) and bodily pain (29.1). Improvements were sustained throughout the study during treatment with canakinumab.

HRQoL for the five pediatric patients was assessed by using the CHQ-PF28. A mean CHQ-PF28 physical summary score at baseline of 43.4 indicated a reduced quality of life compared with that expected for normal children in the United States (that is, 50.0). At the end of part 1, the mean score was 53.4, and the score was 50.1 at the end of part 3. Mean CHQ-PF28 psychosocial score was 54.3 at baseline and showed a small improvement over the course of the study to 56.1 at the end of part 3.

### Safety and tolerability

As previously reported [6], therapy with canakinumab for up to 48 weeks was generally well tolerated. Only two serious AEs were reported: recurrent antibiotic-resistant lower urinary tract infection and sepsis in one patient; and vertigo and increased intraocular pressure, acute glaucoma, and unilateral blindness (complications of CAPS) in a second patient. Only one patient (the former) discontinued because of an AE.

## Discussion

Most patients included in this study had MWS, a moderately severe form of CAPS, and four patients had more-severe disease. This was reflected in the severity of symptoms at baseline, with almost all patients having arthralgia, conjunctivitis, urticarial skin rash, headache, and fatigue. In addition, myalgia, pyrexia, and deafness were reported in more than 50% of patients. In this study, we report the effects of canakinumab on these CAPS-related symptoms (except for amyloidosis, for which the duration of follow-up was insufficient for significant changes to be observed, and deafness, for which the potential for significant recovery of hearing has not been established). These results are in agreement with previous reports of the symptoms associated with the more-severe forms of CAPS [13,14], and give a measure of how debilitating this disorder is and the need for effective treatment.

The results of this study indicate that a single dose of canakinumab induces rapid remission of symptoms in patients with CAPS; on day 8 (the first assessment), 89% of patients had no or minimal disease activity, according to the physician global assessment, and 82% of patients rated their symptoms as absent or minimal. Rapid remission was observed for all individual symptoms of CAPS assessed, and responses were sustained at week 8, the end of part 1 of the study. Remission of symptoms was accompanied by suppression of inflammation, as indicated by decreases in median levels of CRP, SAA, and IL-6. Furthermore, decreases in platelet counts and WBC counts and increases in hemoglobin levels were observed over the course of part 1 of the study, consistent with suppression of chronic inflammation.

Effective suppression of inflammation and symptoms of CAPS was maintained during treatment with canakinumab every 8 weeks during parts 2 and 3 of the study. At the end of part 3, 97% of all patients had no or minimal disease activity; the median IL-6 level was low (< 1 pg/ml); and CRP and SAA levels were below the ULN. For patients randomized to placebo, symptoms returned during part 2, with only four (25%) patients having no or minimal disease activity at the end of part 2. In addition, median IL-6, CRP, and SAA levels increased over the course of part 2. On resuming canakinumab in part 3, remissions were regained in all patients from the placebo group, and CRP and SAA levels returned to levels similar to those seen in patients randomized to canakinumab in part 2.

CAPS can have a profound impact on HRQoL, as was previously demonstrated in a study involving in-depth interviews with patients with FCAS, the mildest form of this disorder [3]. In this study in patients with FCAS, patients reported that their disease had affected their employment, with one third reporting that they had left their job because of the need to accommodate their condition, and more than 95% reported to have limited participation in or avoided outdoor activities and sports. Subjects also reported that their disease had adverse effects on social activities, including socializing and personal relationships.

In our study, we used validated questionnaires to assess in detail the impact of CAPS on HRQoL in pediatric and adult patients, and the changes in HRQoL observed in response to canakinumab therapy. At baseline, SF-36 PCS and MCS, as well as FACIT-F scores, were all below those of the U.S. general population, as we previously reported for a phase 2 study with canakinumab [15]. In addition, HRQoL scores for our patient population were comparable to or well below those reported for patients with musculoskeletal diseases, including rheumatoid arthritis, gout, osteoporosis, and fibromyalgia, in a recent survey of the Dutch general population [16]. Results suggest that the individual SF-36 domains most affected by CAPS in adults are role-physical, bodily pain, and general health, as would be expected from the nature of the symptoms.

Within 8 days of receiving canakinumab, improvements were evident in all SF-36 domains and especially in those that were more than 20 points lower than those of the general U.S. population at baseline. This is in agreement with the rapid remission of symptoms observed in this study. Eight weeks after receiving the first dose of canakinumab, scores for FACIT-F, SF-36 PCS, and SF-36 MCS approached or exceeded scores for the U.S. general population, and improvements were mostly sustained throughout therapy with canakinumab. Moreover, further improvements in scores for all the SF-36 individual domains were observed at 8 weeks after the dose, compared with day 8. These results are in agreement with those reported for the phase 2 study, in which scores for SF-36 PCS, SF-36 MCS, and FACIT-F all approached or exceeded those of the general population by 5 weeks after the dose, and considerable improvements over baseline for all measures were observed at 1 week after the dose [15]. Reductions in functional disability were also observed over the course of the study.

HRQoL results for the five pediatric patients included in the present study also demonstrated an improvement over the course of the study. In these patients, the physical component of HRQoL was well below that expected for healthy children at baseline and exceeded that of healthy children at the end of part 1 and at the end of the study. Although these data are based on only a small patient population, they suggest that the HRQoL benefits reported for adults are also achieved in pediatric patients.

As reported previously [6], the results of this study indicate that canakinumab is generally well tolerated and is not associated with an increased risk of any particular AE, other than a slightly increased risk of infections. Only one patient discontinued the study owing to AEs, and only two serious AEs were reported during the course of the study. Because it is envisaged that patients with CAPS will require life-long therapy, long-term follow-up of patients treated with canakinumab is required to assess more fully the safety profile of this therapy.

This study has a number of limitations. First, the physician and patient global assessments and assessments of the individual symptoms used are not standardized, and hence results cannot be compared with those from other studies that have used different measures. Second, symptoms were assessed on a 5-point scale, which may bias results because it is difficult to detect small variations by using such a scale. Third, the patient's and physician's assessments of severity of symptoms scales are not validated instruments in this disease, which might have led to the differences in results observed between the two at baseline.

The dramatic resolution of symptoms and improvements in HRQoL observed in this study in patients with CAPS is tremendously encouraging and raises the possibility that IL-1β blockade may also benefit patients with other hereditary autoinflammatory disorders in which overproduction of IL-1β is implicated in the pathology. This is supported by promising results that have been reported for IL-1β blockade in individual patients with various autoinflammatory disorders and warrants further investigation [17].

## Conclusions

In summary, the results reported here provide further evidence to indicate that canakinumab induces rapid remission of clinical symptoms of CAPS and suppression of markers of inflammation after administration of a single dose. Remissions were sustained with 8-weekly dosing and led to considerable improvements in HRQoL scores, which approached or exceeded those of the U.S. general population at 8 weeks after the dose and were maintained during canakinumab therapy. Therapy was generally well tolerated, and no immunogenicity was observed. Long-term follow-up of patients is required to confirm the favorable safety profile reported in this 48-week study, and follow-up of children during the next decade will help to determine whether canakinumab therapy can prevent the development of the neurologic complications associated with this disorder.

## Abbreviations

AEs: Adverse events; BP: bodily pain; CAPS: cryopyrin-associated periodic syndrome; CHQ-PF28: Child Health Questionnaire-Parent Form 28; CINCA: chronic infantile neurologic cutaneous and articular syndrome; CRP: C-reactive protein; FACIT-F: functional assessment of chronic illness therapy-fatigue; FCAS: familial cold autoinflammatory syndrome; GH: general health; HAQ: Health Assessment Questionnaire; HRQoL: Health-related Quality of Life; IL-1β: interleukin-1β; IL-6: interleukin-6; MCS: mental-component summary score; MH: mental health; MWS: Muckle-Wells syndrome; NALP3: NACHT, LRR, and PYD domains-containing protein 3; NLRP3: Nod-like receptor protein 3; NOMID: neonatal-onset multisystem inflammatory disease; PCS: physical-component summary score; PF: physical functioning; RE: Role-emotional; RF: Role-physical; SAA: serum amyloid-A protein; SF: social functioning; SF-36: Short-Form Health Survey; VT: vitality; ULN: upper limit of normal.

## Competing interests

Dr Koné-Paut reports having received an honorarium from Novartis for participation in a workshop on canakinumab, research fees from SOBI biovitrum, and consulting fees from Roche, Abbott, BMS, Pfizer, Genzyme, and Novartis. Dr Lachmann reports having received consulting fees from Novartis; Dr Kuemmerle-Deschner reports having received consulting fees from Novartis, Bristol-Meyers Squibb, and Roche and grant support from Novartis. Dr Hachulla reports having received consulting fees and grant support from Novartis. Dr Leslie reports having received consulting fees from Novartis. Dr Mouy reports having received an honorarium from Novartis for participation in this study. Drs Preiss, Ferreira, and Lheritier and Ms Patel are employees of Novartis and report having equity interests in Novartis. Dr Hawkins reports having received consulting fees from Novartis.

## Authors' contributions

IK-P, JB K-D, EH, KSL, and RM were investigators in the study and contributed to data collection. HJL and PNH were investigators and were involved in study design and data collection. NP and KL were in involved in study design and RP and AF in data interpretation. All authors vouch for the accuracy of the data and the analysis, contributed to the interpretation of the data and, were involved in the decision to publish. Medical writing support was provided by Rowena Hughes, Oxford Pharmagenesis Ltd, after teleconferences with all authors, during which the data, its interpretation, and the content of the article were discussed. All authors approved the submitted manuscript.

## Supplementary Material

Additional file 1 **Supplementary file 1: **The file contains supplementary information on patient's demographics, study design, and patient's disposition. The file contains one table: Demographics and baseline disease characteristics. showing patients ages, sex, types of NLRP3 mutations and previous anti IL-1 treatments. **Supplementary Figure 1**. Study design: showing in details the three parts of the study: part 1, open-label 8 weeks; part 2, withdrawal period, double-blinded placebo-controlled study; and part 3, open-label period. **Supplementary Figure 2**. Patient disposition: showing the distribution of patients from assessment eligibility to enrollment, and then through different phases of treatment and study termination. Click here for file
