# Supplementary material for: Sustained remission of symptoms and improved health-related quality of life in patients with cryopyrin-associated periodic syndrome treated with canakinumab: results of a double-blind placebo-controlled randomized withdrawal study
Source: Arthritis Res Ther. 2011 Dec 9;13(6):R202. doi: 10.1186/ar3535 (PMC3334655; doi:10.1186/ar3535)

Supplementary information

Supplementary table. Demographics and baseline disease characteristics

**Variables**	**Patients (n = 35)**
Age, years	
Mean ± SD	34.0 ± 14.9
Median (range)	36.0 (9–74)
Female, n (%)	25 (71.4)
Caucasians, n (%)	33 (94.3)
Form of CAPS, n (%)	
MWS	33 (94.3)
MWS/NOMID	4 (11.4)
NLRP3 mutation, n (%)	
R260W	18 (51.4)
T348M	7 (20.0)
D303N	3 (8.6)
E311K	2 (5.7)
Other*	5 (14.3)
Previous treatment, n (%)	
Canakinumab	9 (25.7)
Anakinra	17 (48.6)

*One each of M662T, A439V, D305N, T436N, and T436I.

CAPS, cryopyrin-associated periodic syndrome; MWS, Muckle–Wells syndrome; NOMID, neonatal-onset multisystem inflammatory disease.

Supplementary figure 1. Study design

The time point for entering part 3 was based on completion of part 2 or relapse.

*For patients who completed part 2 and part 3 had a duration of 16 weeks, with patients receiving two injections of canakinumab. For patients who relapsed in part 2 and part 3 had a duration of up to 40 weeks, with patients receiving canakinumab injections every 8 weeks.

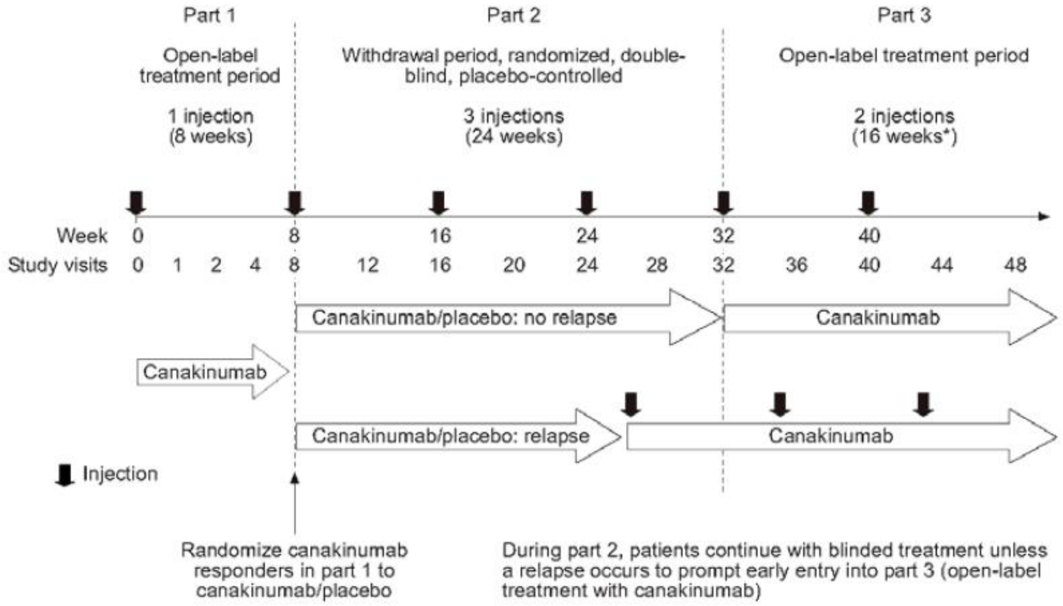

Supplementary figure 2. Patient disposition

CR, complete response; ITT, intent-to-treat.

* one patient relapsed on the last day of part 2

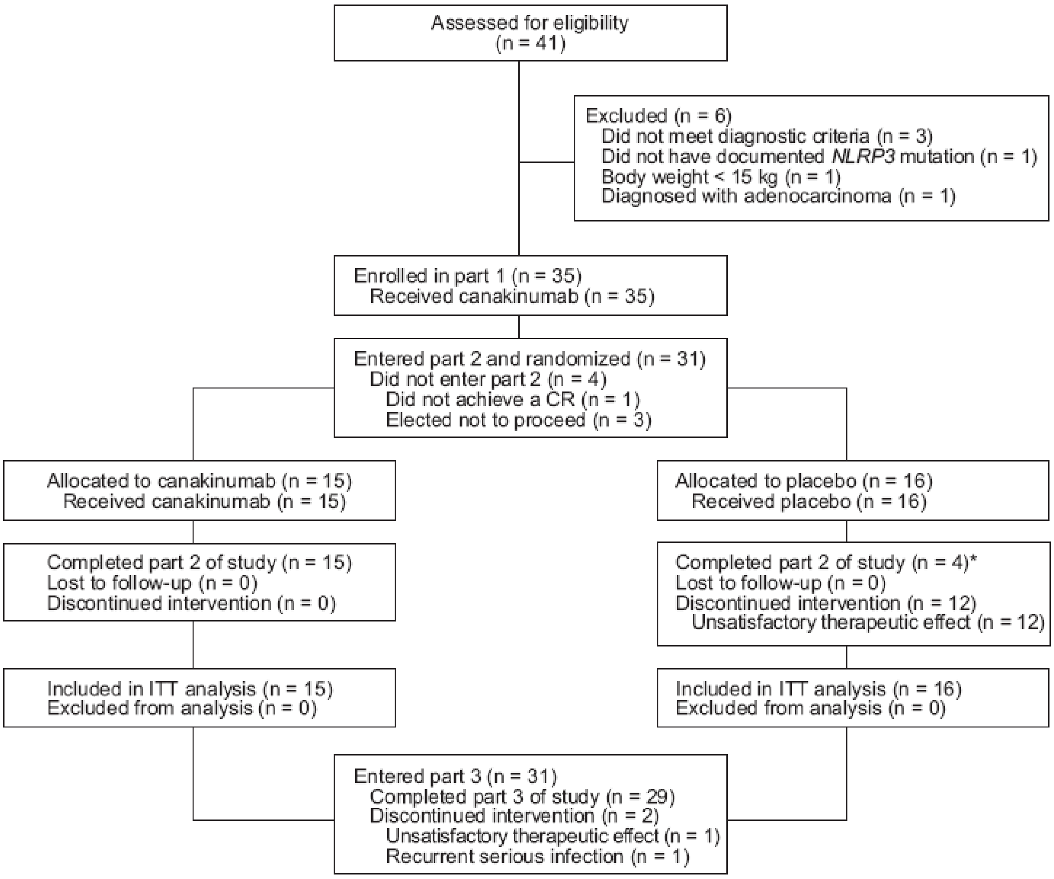

Supplement: Additional file 1 — Supplementary file 1: The file contains supplementary information on patient's demographics, study design, and patient's disposition. The file contains one table: Demographics and baseline disease characteristics. showing patients ages, sex, types of NLRP3 mutations and previous anti IL-1 treatments. Supplementary Figure 1. Study design: showing in details the three parts of the study: part 1, open-label 8 weeks; part 2, withdrawal period, double-blinded placebo-controlled study; and part 3, open-label period. Supplementary Figure 2. Patient disposition: showing the distribution of patients from assessment eligibility to enrollment, and then through different phases of treatment and study termination. [file ar3535-S1.PDF]
